# Fatty Acid Profile and Cardiometabolic Markers in Relation with Diet Type and Omega-3 Supplementation in Spanish Vegetarians

**DOI:** 10.3390/nu11071659

**Published:** 2019-07-20

**Authors:** Ana M. Salvador, Elena García-Maldonado, Angélica Gallego-Narbón, Belén Zapatera, M. Pilar Vaquero

**Affiliations:** Department of Metabolism and Nutrition, Institute of Food Science, Technology and Nutrition (ICTAN-CSIC), José Antonio Novais, 10, 28040 Madrid, Spain

**Keywords:** omega-3 fatty acids, *n*-6/*n*-3 ratio, cardiometabolic markers, homocysteine, vegetarian, vegan, supplementation, oleic acid, alpha-linolenic acid, docosahexaenoic acid

## Abstract

Plant-based diets are becoming increasingly popular, and scientific information concerning the nutritional status in this population is needed. This study determined the fatty acid profile of Spanish lacto-ovo vegetarians (LO-vegetarians) and vegans. Participants were 104 healthy adults, LO-vegetarians (*n* = 49) and vegans (*n* = 55). Lifestyle habits and consumption of food and omega-3 supplements were estimated by questionnaires. BMI, blood pressure, and abdominal and body fat were determined. Serum was collected to analyze fatty acids, glucose, lipids, homocysteine, insulin, and leptin. Volunteers were classified according to serum omega-6 to omega-3 (*n*-6/*n*-3) ratio into three groups: *n*-6/*n*-3 < 10, *n*-6/*n*-3 ≥ 10 to 20, and *n*-6/*n*-3 > 20. Results showed low cardiovascular risk and high insulin sensitivity with negligible differences between diet types. Linoleic acid (C18:2*n*-6) was the major serum fatty acid, followed by oleic (C18:1*n*-9) and palmitic (C16:0) acids. In contrast, serum eicosapentaenoic acid (EPA, C20:5*n*-3) and docosahexaenoic acid (DHA, C22:6*n*-3) were (median, interquartile range) 0.27, 0.18% and 1.59, and 0.93%, respectively. Users of *n*-3 supplements (<10% of total vegetarians) had significantly higher EPA than non-users, while frequent consumption of flax-seeds was associated with increased α-linolenic acid (C18:3*n*-3). However, neither *n*-3 supplementation nor food consumption affected DHA levels in this vegetarian population.

## 1. Introduction

Diets exclusively based on plant foods have become progressively common in developed countries. However, the scientific evidence concerning the nutritional status of this population is limited. Vegetarian diets are usually associated with the prevention of some diseases such as ischemic heart disease, obesity and type 2 diabetes [1]. Nevertheless, they may result in insufficient intake of several nutrients such vitamin B12, vitamin D, and essential fatty acids [2,3]. There are two essential polyunsaturated fatty acids (PUFAs) for human nutrition: linoleic acid (LA, C18:2*n*-6) and α-linolenic acid (ALA, C18:3*n*-3), of the *n*-6 and *n*-3 PUFAs pathways respectively. These fatty acids are substrates for long-chain PUFAs which exert important functions. LA is a precursor of arachidonic acid (AA, C20:4*n*-6), and ALA of eicosapentaenoic acid (EPA, C20:5*n*-3) and docosahexaenoic acid (DHA, C22:6*n*-3) [3]. In addition, the eicosanoids derived from the *n*-6 or *n*-3 pathways have pro-inflammatory or anti-inflammatory activities, respectively, [4] and these should be balanced for adequate health maintenance.

Vegetarian diets, of which the best characterized are lacto-ovo vegetarian (LO-vegetarian) and vegan, provide high intakes of *n*-6 but are low in *n*-3 PUFAs, as the principal dietary source of EPA and DHA is oily fish, which is absent in vegetarian diets. In this line, it has been observed that vegetarians have lower plasma concentrations of both fatty acids compared to omnivores [5], and there may be relevant health implications since these fatty acids are crucial for brain and retina development, and exert important neurological, anti-inflammatory, antithrombotic, and cognitive functions [6]. In this regard, it has been reported that Western diets have ratios of *n*-6/*n*-3 in the range of 15–20 and a ratio lower than 10 has been proposed [7], though there is no consensus on this issue [8,9].

Assessment of fatty acids intake can be based on dietary questionnaires and food composition databases. However, information on the composition of new foods and vegetarian foods is lacking or incomplete, which limits the applicability of this procedure. Currently, the use of intake biomarkers is preferred. Therefore, in the present study, serum fatty acids were analyzed as short-term intake markers [10,11].

Given that there is no available research on the relationship between plant-based diets and health biomarkers of Spanish vegetarians, the objectives of this study are: (1) to determine the fatty acid profile and cardiometabolic markers of Spanish vegetarians and characterize the possible differences between LO-vegetarian and vegans; (2) to evaluate whether this vegetarian population uses *n*-3 supplements or not and their effect on serum fatty acids; and (3) to determine if there are differences in cardiometabolic biomarkers according to *n*-6/*n*-3 ratio and the contribution of fat rich foods.

## 2. Materials and Methods 

### 2.1. Study Design and Subjects 

This study is part of a wider research with a cross-sectional design. Recruitment was performed in the area of Madrid, Spain, through different advertisements in web pages and social networks inviting healthy LO-vegetarian or vegan adults (age ≥18 years) to participate in this research. Exclusion criteria were: occasional meat or fish consumption, diagnosed digestive, renal, hematologic, endocrine or oncological diseases, eating disorders, pregnancy, lactation, and menopause. Initially, a total of 194 subjects were interested, of which 56 were excluded and 33 declined to participate. Finally, a total of 105 volunteers were selected for the study, of which 104 participated and gave consent to blood extraction (81 women and 23 men).

This study was conducted in accordance with the ethical principles expressed in the Declaration of Helsinki and was approved by the Clinical Research Ethics Committee of Puerta de Hierro University Hospital (Madrid) and the Ethics Committee of the Spanish National Research Council (CSIC). A written informed consent was obtained from all the participants.

### 2.2. Dietary Assessment, Anthropometric and Body Composition Measurements

Volunteers were asked to fill out an on-line lifestyle and food frequency questionnaires (FFQ) previously used by our research group [6]. Diet type, LO-vegetarian or vegan, consumption of *n*-3 supplements, smoking habits, physical activity, and educational level were assessed. Food consumption was classified from 0 to 5 according to the following frequency categories: once a month or less (0), two to four times a month (1), two to three times a week (2), four to six times a week (3), once a day (4), and twice a day or more (5). Supplement intake was classified from 0 to 4 as follows: never (0), one to 12 times a year (1), two to five times a month (2), two to six times a week (3) and daily (4). Food items being fat sources were considered, including nuts (walnuts, cashew nuts, almonds, peanuts, pistachio, soy nuts, pecans, Brazil nuts, and hazelnuts), seeds (pumpkin seeds, sesame seeds, sunflower seeds, flax-seeds, and poppy seeds), nut butters (cashew butter, peanut butter, almond butter, soy nut butter, and tahini), oils (olive oil, sunflower oil, soybean oil and canola oil), and others (avocado and olives). 

Volunteers’ height, body weight, and waist and hip perimeters were measured. Body mass index (BMI) and body composition were obtained (Tanita BC-601, Tanita Ltd., Amsterdam, Netherlands).

### 2.3. Blood Sampling and Cardiometabolic Markers Determinations

Blood samples were collected in Vacuette Z Serum Sep Clot Activator tubes (Greiner Bio-One GmbH, Frickenhausen, Germany) at the Human Nutrition Unit (UNH) of the Institute of Food Science, Technology and Nutrition (ICTAN-CSIC, Madrid, Spain) from 8:00 h to 8:30 h, after a 12 h fasting overnight period. Serum was isolated by centrifugation in a Jouan CR-312 centrifuge (Jouan Ltd, Ilkeston, UK) at 1000 g for 15 min and samples were then stored at −30 °C for fatty acid analysis. Fatty acids were measured in serum because serum fatty acid levels are correlated with recent intake and reflect the dietary habits of the subjects at the time of the study [10,11]. Moreover, elaidic acid (ELA, C18:1*n*-9t) was analyzed as an unsaturated trans-fatty acids status indicator because it is the major trans-fatty acid found in hydrogenated vegetable oils.

Serum glucose, total cholesterol (TC), HDL-cholesterol (HDLc), LDL-cholesterol (LDLc), and triglycerides (TAG) were analyzed in the ADVIA Chemistry XPT System (Siemens, Erlangen, Germany). Homocysteine (Hcy) was analyzed by competitive immunoassay of direct chemiluminescent technology in an ADVIA Centaur XP autoanalyzer (Siemens). Insulin and leptin were determined by ELISA kits (DRG Instruments, Marburg, Germany). Blood pressure was measured using an automated digital oscillometric device (Omron model M6 Comfort, Omron Corporation, Tokyo, Japan). Insulin resistance (HOMA-IR) was estimated by the homeostasis model assessment index as [glucose (mg/dL) × insulin (µU/mL)]/405; and insulin sensitivity, by the quantitative insulin sensitivity check index (QUICKI) as 1/[log insulin (µU/mL) + log glucose (mg/dL)].

### 2.4. Serum Fatty Acid Analysis

Fatty acids analysis was performed by gas chromatography (GC) using a Varian CP-3800 gas chromatograph with a flame ionization detector (Varian, Inc., Palo Alto, CA, USA). Fatty acid methyl esters (FAMEs) were obtained based on the method developed by Lepage and Roy [12]. Two mL of a 4:1 (v/v) methanol:benzene solution were added to 200 μL of serum samples plus 25 μL of methyl heptadecanoate, the internal standard. After mixing carefully, 200 μL of acetyl chloride were slowly incorporated while stirring. Tubes were firmly closed and subjected to methanolysis at 100 °C for 1 h. Once tubes were cooled, 5 mL of potassium carbonate solution (6% w/v) were added to stop the reaction and to neutralize the mixture. Tubes were stirred and centrifuged (1000 g, 10 min) to separate the two resulting phases. The upper phase was transferred to GC vials and injected into the gas chromatograph. The separation of the FAMEs was carried out on a BPX70 capillary column (30 × 0.25 × 0.25) from SGE Analytical Science (Melbourne, Australia). Fatty acid peak areas and concentrations were determined using the Empover 3 software (Waters Corporation, Milford, MA, USA). For statistical analyses, data were expressed as percentage of total fatty acids.

The following saturated fatty acids (SFA) were analyzed: myristic acid (MIR, C14:0), palmitic acid (PAL, C16:0), and stearic acid (STE, C18:0). Measured monounsaturated fatty acids (MUFA) were: ELA, palmitoleic acid (POA, C16:1*n*-7), and oleic acid (OA, C18:1*n*-9). Finally, analyzed PUFA were: LA, dihommo γ-linolenic acid (DGLA, C20:3*n*-6), AA, γ-linolenic acid (GLA, C18:3*n*-6), ALA, EPA, and DHA. 

### 2.5. Statistical Analysis

The distribution of the variables was analyzed by the Kolmogorov-Smirnov test, and natural log-transformed data were used for statistical analysis when it was possible. However, several variables could not be normalized: STE, ELA, OA, MUFA, *n*-6, and the ratios OA/STE, LA/OA, ALA/LA, GLA/LA, EPA/ALA, DHA/AA, PUFA/SFA, and PUFA/MUFA. The data were expressed as median (interquartile range), except for the proportion of subjects in each gender; age; *n*-3 supplementation; smoking habits; education level; and physical activity groups, which were expressed as *n* (%).

Differences between diet type groups were studied by independent samples *t*-test or Mann-Whitney’s *U* test for normally distributed and non-normally distributed variables, respectively. The studied subjects were classified into three groups according to the following cut-off levels: *n*-6/ *n*-3 ≤ 10; *n*-6/*n*-3 > 10 and ≤20; *n*-6/*n*-3 > 20. Differences among them were studied by χ^2^ tests or Kruskal-Wallis tests. The significance level was set at *p* < 0.05, and all the statistical analyses were performed with SPSS 24.0 for Windows (IBM, Armonk, NY, USA).

## 3. Results

### 3.1. Characteristics of the Participants

Characteristics of the studied population are described in Table 1. The study was completed by 104 volunteers, of which 49 were LO-vegetarians (11 men and 38 women) and 55 were vegans (12 men and 43 women). The majority of the studied individuals were young, with a median age of 30 years, non-smokers, had a bachelor’s degree, and exhibited moderate physical activity. Only nine volunteers were *n*-3 supplement users (four LO-vegetarians and five vegans). There were no significant differences between LO-vegetarians and vegans in terms of BMI, waist circumference, body fat, abdominal fat, glucose, insulin, leptin, Hcy, TC, HDLc, LDLc, TAG, and cardiometabolic indexes (TC/HDLc, LDLc/HDLc. TAG/HDLc, HOMA-IR, QUICKI, and HOMA-B). SBP was similar in both groups, although DBP was slightly lower in vegans than in LO-vegetarians (*p* = 0.047). 

### 3.2. Serum Fatty Acids and Main Cardiometabolic Indexes

Total serum fatty acids for the whole population (median, interquartile range) were 2005.8 and 607.6 µg/mL, without significant differences between women and men. Table 2 presents the percentiles of serum fatty acids percentage. Major serum fatty acids were LA, OA and PAL. In contrast, the trans-fatty acid status indicator ELA was very low. Table 3 shows the differences of the types of vegetarian diets and *n*-3 supplementation on serum fatty acids. Vegans compared with LO-vegetarians had significantly higher OA (*p* = 0.010), OA/STE (*p* = 0.047), MUFA (*p* = 0.018), and AA/(EPA+DHA) (*p* = 0.026), but lower ELA (*p* = 0.003) and *n*-3 (*p* = 0.048) levels. All other parameters were not significantly different between the two vegetarian diet groups.

Users of *n*-3 supplements showed higher total *n*-3 (*p* = 0.045), EPA (*p* = 0.024) EPA/AA (*p* = 0.026), EPA/ALA (*p* = 0.028), and lower *n*-6/*n*-3 (*p* = 0.039) compared to non-users. No other significant differences between *n*-3 supplement users and non-users were observed.

### 3.3. LO-vegetarians and Vegans Classification According to n-6/n-3 Ratio

Volunteers were classified into three groups according to their n6/n3 ratio: <10, ≥10 to 20, and >20 (Table 4). Gender distribution neither varied among groups nor were there any differences in physical activity or anthropometric and body fat parameters. There were significantly higher proportions of LO-vegetarians and *n*-3 supplement users (*p* = 0.032 and 0.017, respectively, χ^2^ test) in the lowest and middle *n*-6/*n*-3 groups compared to the *n*-6/*n*-3 > 20 group. Concerning the cardiometabolic markers, no significant differences among *n*-6/*n*-3 groups were observed for glucose, lipid and hormone levels (insulin, leptin), but serum Hcy was significantly lower in the *n*-6/*n*-3 < 10 group than in the other groups (*p* = 0.003). The level of the monounsaturated fatty acid POA was higher in the *n*6/*n*3 < 10 group (*p* = 0.024) while the *n*-6 LA and the *n*-3 fatty acids ALA, EPA and DHA were the main contributors to the differences among the *n*-6/*n*-3 groups. 

**Figure 1:**
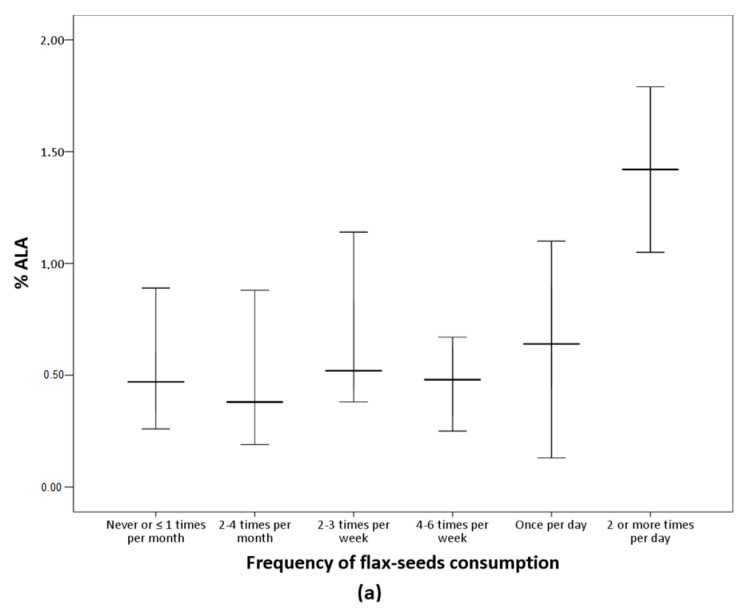

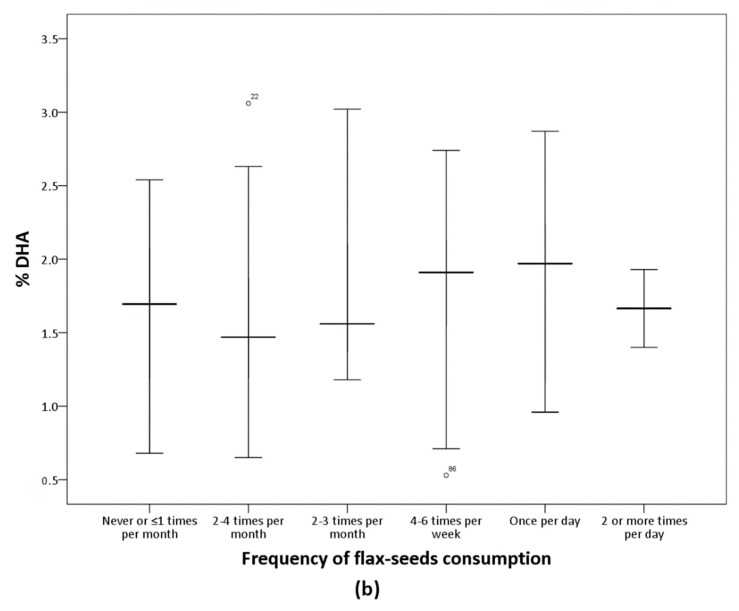
Percentage of ALA and DHA according to flax-seeds frequency of consumption. Median values and interquartile ranges are presented. (**a**) Significant differences among groups by Kruskal-Wallis (*p* = 0.001). (**b**) The differences among groups were not significant (*p* = 0.145).

The analysis of the FFQ data confirms that participants in the LO-vegetarian group consumed milk, eggs and their derived products while vegans did not declare any consumption of these products. Concerning the culinary fat, most of the vegetarians consumed olive oil several times per day (Table 5). Table 5 shows the frequency of consumption of nuts, seeds, oils, and other fat-rich foods of the subjects classified according to *n*-6/*n*-3 groups. Flax-seeds were more frequently consumed in the *n*-6/*n*-3 < 10 group but the differences were marginally significant (*p* = 0.056) and no other differences were observed. Figure 1 presents serum ALA and DHA according to frequency of consumption of flax-seeds. Serum ALA increases as flax-seeds intake increases while no variation in serum DHA was observed in relation to the consumption of this food item.

## 4. Discussion

In this study, serum fatty acids profiles of Spanish vegetarians are presented for the first time. Participants were healthy young adults, with similar sample sizes of LO-vegetarians and vegans, and less than 10% of the volunteers consumed *n*-3 supplements. Lifestyle habits, body composition, physical activity, and cardiometabolic markers indicate that this population has low cardiovascular risk and high insulin sensibility. 

The major serum fatty acid was LA, followed by OA and PAL; while levels of *n*-3 PUFA, except for ALA, were low in comparison with the data reported for general population [13]. These results clearly show an inadequate *n*-6/*n*-3 ratio, indicating a metabolic imbalance. While the results of LA and *n*-3 levels are consistent with other reports of vegetarians [5,14], OA levels were remarkably high in comparison to other countries, representing about 20–25% of the total serum fatty acids. For instance, OA values of vegetarians in China, South India and Australia were around 1%, 7%, and 10%, respectively [15,16,17]. This is certainly due to the high olive oil consumption characteristic of the Mediterranean diet in Spain. Consistently, this oil was consumed by the volunteers several times per day (Table 5) and our OA levels were similar to those of the Spanish PREDIMED study at baseline point [13,18]. 

Moreover, vegans presented higher OA (and consistently higher MUFA and OA/STE) and lower ELA, the trans isomer of OA, compared to LO-vegetarians. This trans fatty acid is a marker of ultra-processed food, e.g., margarine, chocolate, potato flakes, potato fries, breakfast cereals, etc. [19], which would suggest a more unfavorable diet in LO-vegetarians than vegans. However, the values obtained in our vegetarian population, either LO-vegetarians or vegans, were much lower than those of other European populations. In this regard, plasma ELA was about three times higher in the EPIC cohort [20,21]. The results of the FFQ of the present study confirm that the frequency of consumption of industrial processed food was similar in LO-vegetarians and vegans, except for food items that contained eggs and milk whose consumption was declared null by the vegans. Natural ELA is present in ruminant’s milk, and thus the consumption of milk and dairy products may have contributed to the slightly higher ELA in LO-vegetarians in comparison with vegans [22]. Nevertheless, the generally low ELA levels obtained in the present study suggest a low consumption of ultra-processed food in both LO-vegetarians and vegans.

Vegans presented lower *n*-3 levels than LO-vegetarians, which can be mainly attributed to their lower percentage of DHA; but the fatty acid profiles did not markedly differ between these groups. In fact, and consistently with other studies in vegetarians [5], PUFAs were near 50% of all fatty acids, and LA (C18:2*n*6) was the major PUFA independently of subject’s classification. Therefore, there was an imbalance in *n*-6/*n*-3, except for the subjects who consumed supplements of *n*-3 fatty acids (Table 3). 

The metabolic changes induced by the elimination of the main sources of *n*-3 PUFA, fish and their respective products, from the diet should be briefly commented. It has been demonstrated that the *n*-6 and *n*-3 pathways are interconnected. The essential fatty acids LA and ALA are precursors of the *n*-6 and *n*-3 PUFA families, respectively. These fatty acids compete for the same enzymes Δ6 and Δ5 desaturases and elongases, and the Δ6 desaturase constitutes the rate-limiting enzyme, presenting higher affinity for ALA than LA [23]. However, under the high LA consumption conditions observed in the present study, the *n*-6 pathway predominates in detriment of the *n*-3 pathway [24]. Consistently, the observed ALA/LA ratios indicate that ALA levels (*n*-3) were approximately 100 times lower than those of LA (*n*-6). Moreover, results of EPA/ALA and DHA/ALA confirm the low intake of both EPA and DHA, typical of vegetarian diets, suggesting that the production of these fatty acids from ALA was very limited. In agreement, the overall calculated efficiency of conversion from ALA to DHA is lower than 1% or even 0.01%, depending on the used models [23,25,26]. Interestingly, consumption of *n*-3 supplements (by less than 10% of the total volunteers) was associated with an increase of the EPA levels and the EPA/ALA and EPA/AA ratios without a parallel rise in DHA. This can be explained by the poor enzymatic efficiency in the DHA production from EPA and ALA, and by the composition of the *n*-3 supplements consumed. In this line, Brenna et al. summarized the change in blood *n*-3 PUFAs after the consumption of supplements containing ALA or EPA [27], and concluded that the intake of these fatty acids cannot increase serum DHA. Therefore, present results reflect an inadequate supplementation practice. We observed that the *n*-3 supplements used were very diverse, ranging from supplements containing DHA specially designed for vegetarians to EPA+DHA combinations manufactured from fish or mixtures of *n*-3, *n*-6, *n*-9, and *n*-7. Indeed, when volunteers were classified according to the *n*-6/*n*-3 ratio, only one third of the subjects included in the *n*-6/*n*-3 < 10 were *n*-3 supplements users.

The cardiometabolic risk markers (glucose, lipids, insulin, the insulin resistance/sensitivity indexes, leptin and Hcy) studied according to the *n*-6/*n*-3 classification, confirm that this vegetarian population exhibits a low cardiometabolic risk, in agreement with other authors [28,29]. Moreover, Hcy, which constitutes an independent factor for cardiovascular disease [30], was lower in the *n*-6/*n*-3 < 10 group compared to the others. This result cannot be attributed to vitamin B12 deficiency, since we confirmed that 75% of the participants consumed vitamin B12 supplements, as previously described [3,31], and vitamin B12-supplemented subjects were equally distributed among the *n*-6/*n*-3 groups. In fact, an inverse relationship between *n*-3 and Hcy has been observed in different studies [17,32]. Bertasd et al. [33] observed that self-reported fish intake was negatively associated with Hcy plasma concentrations in a large cohort of Norwegian people, and another study found that *n*-3 supplement consumption (3,6 g/day) during 3 months, decreased Hcy in treated dyslipemic diabetics in comparison with a placebo [34].

The higher POA in the group *n*-6/*n*-3 < 10 may be related to a higher intake of this fatty acid or may also reflect the Δ9 desaturase activity that converts PAL into POA [35]. In this group, higher ALA, EPA and DHA were shown together with lower *n*-6 PUFA LA, as expected. Concerning the results of the different fatty acid ratios, lower serum EPA/AA has been related with increased risk of coronary heart disease in a Japanese cohort of adults [36]. In another study, a ratio of EPA/AA lower than 0.40 was associated with adverse cardiac events in coronary patients [37]; and although our population was healthy, the observed results are much lower than this cut-off value. Other ratios such as the DHA/AA appear to be less valuable as markers of cardiovascular risk [38]. Therefore, we conclude that the vegetarians of the present study were at low cardiometabolic risk but most of them have an imbalance *n*-6/*n*-3, and low levels of EPA and particularly DHA.

As the group with *n*-6/*n*-3 < 10 included consumers and non-consumers of *n*-3 supplements, the influence of any specific food item intake on serum long-chain *n*-3 fatty acids was evaluated. We observed similar consumption patterns of nuts and oils in the three *n*-6/*n*-3 groups while among the seeds, results suggest an improvement of the *n*-6/*n*-3 ratio associated with higher frequency of consumption of flax-seeds. In fact, these seeds are rich in ALA, and consistently, results showed higher serum ALA concentration with higher intake of flax-seeds (Figure 1). However, there was no rise in serum DHA as flax-seed intake increased. This is according to studies reporting that the intake of ALA-rich foods is insufficient to increase serum DHA [23,26,27]. Moreover, this coincides with the results discussed above regarding *n*-3 supplementation effects on DHA. Altogether, results suggested that the *n*-3 supplementation increased EPA but not DHA, and the intake of specific foods was insufficient to increase DHA serum levels.

There are many aspects of the relationship between plant-based diets and health that remain unknown. A recent meta-analysis on the effect of vegetarian diets on major cardiovascular outcomes, found very low evidence of associations of these diets with reduction of coronary heart disease incidence (atherosclerosis and/or myocardial infarction) and mortality, but no association with overall cardiovascular disease mortality and stroke mortality [39]. The specific effects of *n*-3 fatty acids on heart and circulatory disease was evaluated in a Cochrane systematic review. The review provides moderate- and high-quality evidence that increasing the intake of long-chain *n*-3 fatty acids, mainly from supplements, does not benefit heart health or reduce the risk of stroke or mortality from any cause, and found low-quality evidence suggesting that increasing plant-based ALA may be slightly protective for some heart and circulatory diseases [40]. However, although studies on vegetarians were not excluded in this review, the available information was very limited.

This opens new research lines, as long-chain *n*-3 fatty acids are relevant in lipid metabolism, lipogenesis and β-oxidation and may be implicated in reducing cardiovascular events. Therefore, further studies are needed to establish if preformed DHA supplements should be widely recommended to vegetarians. At present, ALA and LA are the two fatty acids accepted as essential, but perhaps this should be re-evaluated for the vegetarian and whole population. On one hand, more research should be done on the design of safe and bioavailable DHA sources of plant origin, i.e., marine algae; on the other hand, measurements of cardiometabolic markers should be complemented with functional markers, such as blood hemodynamic, visual and brain function tests, to be used in controlled and well-designed interventions in vegetarian and non-vegetarian volunteers.

This study presents several limitations. The volunteers were LO-vegetarians and vegans but no control group of meat and fish eaters was included. Dietary assessment was carried out by an FFQ and details of quantities of food or nutrients ingested are not known. Apart from that, this is the first study that evaluates lifestyle habits, body composition, cardiometabolic risk markers, and serum fatty acid profile of Spanish vegetarians.

## 5. Conclusions

Fatty acid profile of Spanish LO-vegetarians and vegans is characterized by high levels of LA and OA, as olive oil is widely consumed in this Mediterranean population. However, levels of long-chain *n*-3 fatty acids are very low, and consumption of *n*-3 supplements is associated with an increase in EPA but not DHA. Similarly, frequent consumption of ALA-rich seeds is reflected in serum ALA levels, but further conversion to EPA and DHA is undetected. 

## Figures and Tables

**Table 1:** Characteristics of the studied population.

Variables	Total Population	LO-Vegetarians	Vegans
Men/Women, *n*	23/81	11/38	12/43
Age, years	30 (12)	31 (12)	27 (12)
*n*-3 Supplement users, *n* (%)	9 (8.4)	4 (8.2)	5 (9.3)
Smokers, *n* (%)	11 (10.3)	6 (12.2)	5 (9.3)
Educational level, *n* (%)			
Baccalaureate	11 (10.3)	5 (10.2)	6 (11.1)
Bachelor’s degree	54 (50.5)	26 (53.1)	28 (51.9)
Master’s degree and PhD level	36 (35.0)	17 (34.7)	19 (35.2)
Physical activity, *n* (%)			
Low	8 (7.5)	4 (8.2)	4 (7.3)
Moderate	45 (42.1)	20 (40.8)	25 (45.5)
Vigorous	51 (47.7)	25 (51)	26 (47.3)
Anthropometry and body fat			
BMI (kg/m2)	21.95 (3.8)	22.30 (5.4)	21.50 (3.0)
Waist circumference (cm)	77.6 (13)	79.2 (15.8)	77.3 (12.2)
WHR	0.82 (0.09)	0.81 (0.09)	0.82 (0.10)
Body fat (%)	23.8 (11.4)	24.9 (12.4)	23.3 (10.9)
Abdominal fat (%)	19.85 (13.2)	21.8 (14.9)	18.7 (10.4)
Cardiometabolic markers			
SBP (mm Hg)	110.8 (18.8)	112.0 (19.0)	108.5 (19.0)
DBP (mm Hg)	70.2 (12.4)	73.0 (14.5)	69.0 (13.0)
Glucose (mg/dL)	83 (8)	82 (8.0)	84 (9.0)
Insulin (µU/mL)	6.68 (5.27)	6.79 (5.39)	6.50 (4.81)
Leptin (ng/mL)	4.73 (6.13)	5.31 (5.98)	3.64 (6.1)
Hcy (µmol/dL)	12.9 (5.2)	13.3 (5.7)	12.5 (4.9)
TC (mg/dL)	152 (52)	158 (55.0)	145 (52.0
HDLc (mg/dL)	62 (21)	65 (24.0)	60 (20.0)
LDLc (mg/dL)	72.5 (34)	77.0 (38.0)	70.0 (31.0)
TAG (mg/dL)	68 (48)	66 (44.0)	73 (51.0)
TC/HDLc	2.5 (0.7)	2.4 (0.60)	2.5 (0.73)
LDLc/HDLc	1.2 (0.50)	1.2 (0.50)	1.2 (0.63)
TAG/HDLc	0.53 (0.36)	0.44 (0.38)	0.54 (0.38)
HOMA-IR	1.38 (1.14)	1.38 (1.26)	1.40 (0.98)
QUICKI	0.36 (0.04)	0.36 (0.05)	0.36 (0.04)

Results are expressed as *n* (%) or median (interquartile range). SBP: Systolic blood pressure; DBP: Diastolic blood pressure; TC: Total cholesterol; HDLc: High-density lipoprotein cholesterol; LDLc: Low-density lipoprotein cholesterol; TAG: Triacylglycerides; HOMA-IR: Homeostasis model assessment-estimated of insulin resistance; QUICKI: Quantitative insulin sensitivity check index. Differences between diet groups were not significant, except for DBP (*p* = 0.047) (χ^2^, independent samples *t*-tests or Mann-Whitney’s *U* tests).

**Table 2:** Percentiles of fatty acid percentages and main cardiometabolic indexes.

Variables	Percentiles				
	5th	25th	50th	75th	95th
MIR (*C14:0*)	0.25	0.40	0.55	0.74	1.23
PAL (*C16:0*)	15.32	17.57	18.95	20.42	24.00
STE (*C18:0*)	5.78	6.76	7.36	8.13	11.08
ELA (*C18:1n-9t*)	0.02	0.04	0.05	0.08	0.39
POA (*C16:1n-7*)	0.49	0.67	0.97	1.44	2.15
OA (*C18:1n-9*)	16.85	20.16	22.37	24.95	38.10
LA (*C18:2n-6*)	27.34	31.92	34.52	36.85	41.86
DGLA (*C20:3n-6*)	1.18	1.67	2.02	2.40	3.45
AA (*C20:4n-6*)	4.93	7.10	8.16	9.55	11.39
GLA (*C18:3n-6*)	0.21	0.31	0.41	0.57	0.98
ALA (*C18:3n-3*)	0.22	0.34	0.48	0.66	1.09
EPA (*C20:5n-3*)	0.11	0.20	0.27	0.38	0.97
DHA (*C22:6n-3*)	0.78	1.24	1.59	2.17	2.99
TC/HDLc	1.80	2.10	2.45	2.80	3.48
LDLc/HDLc	0.63	1.00	1.20	1.50	2.18
TAG/HDLc	0.24	0.36	0.53	0.72	1.39
HOMA-IR	0.54	0.98	1.38	2.13	4.17
QUICKI	0.31	0.34	0.36	0.38	0.43
WHR	0.72	0.76	0.82	0.86	0.95

Fatty acids are expressed as %. MIR: Myristic acid; PAL: palmitic acid; STE: Stearic acid; ELA: Elaidic acid; POA: Palmitoleic acid; OA: Oleic acid; LA: linoleic acid; DGLA: Dihommo γ-linolenic acid; AA: Arachidonic acid; GLA: γ-linolenic acid; ALA: α-linolenic acid; WHR: Waist-hip ratio.

**Table 3:** Fatty acid profiles according to vegetarian diet type and use of *n*-3 supplements.

Variables	LO-V (*n* = 49)	Vegans (*n* = 55)	*n*-3 Not-Suppl. (*n* = 95)	*n*-3 Suppl. (*n* = 9)	*P*_D_^+^	*P*_S_ *
MIR (%)	0.61 (0.36)	0.53 (0.34)	0.55 (0.30)	0.78 (0.75)	**0.054**	0.148
PAL (%)	19.2 (2.32)	18.65 (2.74)	18.96 (2.75)	18.91 (4.73)	0.088	0.521
STE (%)	7.43 (1.72)	7.35 (1.32)	7.43 (1.50)	7.04 (0.64)	0.166	0.214
ELA (%)	0.07 (0.05)	0.04 (0.03)	0.05 (0.04)	0.07 (0.31)	**0.003**	0.382
POA (%)	1.11 (0.87)	0.86 (0.74)	0.98 (0.73)	0.81 (0.89)	0.255	0.808
OA (%)	21.38 (3.70)	23.89 (5.48)	22.47 (4.68)	21.38 (8.30)	**0.010**	0.742
LA (%)	34.60 (5.12)	34.09 (4.93)	34.60 (4.88)	32.23 (4.80)	0.801	0.124
DGLA (%)	2.04 (0.73)	1.95 (0.74)	1.97 (0.74)	2.13 (0.51)	0.674	0.591
AA (%)	8.19 (1.99)	8.08 (2.59)	8.20 (2.45)	7.81 (2.64)	0.439	0.840
GLA (%)	0.44 (0.25)	0.38 (0.27)	0.40 (0.25)	0.53 (0.31)	0.100	0.334
ALA (%)	0.46 (0.34)	0.49 (0.32)	0.46 (0.33)	0.50 (0.33)	0.906	0.619
EPA (%)	0.30 (0.19)	0.24 (0.17)	0.26 (0.18)	0.49 (0.55)	0.196	**0.024**
DHA (%)	1.85 (0.79)	1.40 (0.93)	1.56 (0.95)	2.09 (1.71)	**0.024**	0.059
DHA/AA	0.2 (0.1)	0.2 (0.1)	0.2 (0.2)	0.2 (0.3)	0.081	0.085
AA/(EPA+DHA)	3.9 (1.65)	4.8 (2.20)	4.2 (2.20)	3.3 (2.85)	**0.026**	0.051
OA/STE	2.9 (1.0)	3.1 (1.1)	3.1 (1.0)	3.4 (1.4)	**0.047**	0.477
LA/OA	1.6 (0.5)	1.5 (0.6)	1.5 (0.5)	1.4 (0.8)	0.075	0.440
ALA/LA	0.01 (0.01)	0.01 (0.01)	0.01 (0.01)	0.02 (0.01)	0.507	0.475
GLA/LA	0.01 (0.01)	0.01 (0.01)	0.01 (0.01)	0.02 (0.02)	0.282	0.108
AA/DGLA	4.2 (2.3)	4.0 (2.2)	4.2 (2.3)	3.9 (1.8)	0.780	0.627
EPA/ALA	0.6 (0.5)	0.6 (0.4)	0.6 (0.4)	1.2 (1.1)	0.087	**0.028**
DHA/ALA	4.28 (3.28)	3.08 (3.15)	3.27 (3.16)	4.22 (3.89)	0.351	0.241
DHA/EPA	6.2 (5.2)	5.6 (5.1)	6.2 (5.4)	4.7 (4.2)	0.808	0.399
EPA/AA	0.04 (0.02)	0.03 (0.03)	0.03 (0.02)	0.06 (0.03)	0.765	**0.026**
SFA (%)	27.72 (4.61)	26.20 (3.87)	26.90 (3.36)	26.75 (6.21)	0.060	0.917
MUFA (%)	22.71 (4.01)	25.15 (5.10)	23.83 (4.70)	23.08 (7.73)	**0.018**	0.720
PUFA (%)	48.72 (5.24)	48.24 (6.26)	48.83 (5.83)	46.71 (5.56)	0.362	0.463
PUFA/SFA	1.8 (0.3)	1.9 (0.40)	1.8 (0.40)	1.8 (0.60)	0.135	0.525
PUFA/MUFA	2.1 (0.60)	2.0 (0.60)	2.0 (0.60)	2.0 (0.80)	0.063	0.610
(PUFA+MUFA)/SFA	2.6 (0.55)	2.8 (0.50)	2.7 (0.40)	2.7 (0.75)	0.060	0.889
*n*-6 (%)	45.90 (5.38)	45.56 (6.54)	45.90 (5.69)	44.39 (2.87)	0.646	0.090
*n*-3 (%)	2.55 (1.13)	2.24 (0.99)	2.40 (0.89)	2.93 (2.18)	**0.048**	**0.045**
*n*-6/*n*-3	17.3 (6.0)	19.9 (8.0)	18.0 (7.7)	11.2 (10.4)	0.107	**0.039**

Results are expressed as median (interquartile range). D: Diet type; S: *n*-3 supplementation. ^+^ Independent samples *t*-tests except for non-normal variables that were analyzed by Mann-Whitney’s U test. * Mann-Whitney’s *U* test. Significant differences are in bold.

**Table 4:** Classification of volunteers according to *n*-6/*n*-3 ratio.

Variables	*n*-6/*n*-3 ≤ 10 (*n* = 9)	10 < *n*-6/*n*-3 ≤ 20 (*n* = 58)	*n*6/*n*3 > 20 (*n* = 37)	*P*-Value *
Women/Men, *n* (%)	8/1 (88.9/11.1)	43/15 (74.1/25.9)	30/7 (81.1/18.9)	0.516
LO-V/vegans *n* (%)	5/4 (55.5/44.4)	33/25 (56.9/43.1)	11/26 (29.7/70.3)	**0.031**
*n*-3 supplemented, *n* (%)	3 (33.3)	5 (8.6)	1 (2.7)	**0.014**
Physical activity, *n* (%)				0.630
Low	1 (11.1)	6 (10.3)	1 (2.7)	
Medium	3 (33.3)	26 (44.8)	16 (43.2)	
Vigorous	5 (55.5)	26 (44.8)	20 (54.1)	
*Anthropometry and body fat*				
BMI (Kg/m^2^)	21.20 (4.4)	22.00 (4.9)	21.80 (2.9)	0.843
Waist circumference (cm)	78.30 (16.8)	78.25 (13.2)	75.10 (11.8)	0.716
Body fat (%)	26.60 (13.5)	24.05 (12.3)	23.30 (10.7)	0.924
Abdominal fat (%)	17.60 (17.1)	19.85 (11.7)	20.00 (12.5)	0.671
*Cardiometabolic markers*				
Glucose (mg/dL)	81.00 (8)	83.50 (9)	83.00 (8)	0.225
Insulin (μU/mL)	6.18 (6.01)	6.61 (4.97)	7.08 (5.65)	0.952
Leptin (ng/mL)	3.30 (5.10)	4.70 (6.16)	5.14 (8.75)	0.386
Hcy (μmol/dL)	9.40 (2.51)	12.70 (5.0)	13.90 (4.2)	**0.003**
TC (mg/dL)	139 (69)	156 (55)	153 (45)	0.825
HDLc (mg/dL)	66 (27)	62 (21)	62 (19)	0.603
LDLc (mg/dL)	51.0 (43)	73.5 (37)	72.0 (29)	0.399
TAG (mg/dL)	74 (100)	80 (55)	64 (43)	0.059
TC/HDLc	1.90 (1.15)	2.50 (0.73)	2.50 (0.70)	0.082
LDLc/HDLc	0.8 (1.0)	1.2 (0.53)	1.2 (0.55)	0.182
TAG/HDLc (mmol/L)	0.49 (0.52)	0.56 (0.38)	0.43 (0.32)	0.082
HOMA-IR	1.31 (1.13)	1.37 (1.16)	1.42 (1.25)	0.855
QUICKI	0.37 (0.05)	0.36 (0.04)	0.36 (0.05)	0.855
*Fatty Acid Percentages*				
MIR	0.64 (0.50)	0.55 (0.37)	0.53 (0.35)	0.187
PAL	19.41 (3.45)	19.16 (3.08)	18.65 (2.70)	0.132
STE	7.10 (2.33)	7.27 (1.35)	7.46 (1.47)	0.374
ELA	0.05 (0.05)	0.05 (0.07)	0.05 (0.03)	0.546
POA	1.31 (1.30)	1.06 (0.77)	0.86 (0.69)	**0.024**
OA	21.25 (7.52)	22.79 (4.98)	21.72 (4.81)	0.736
LA	32.78 (9.30)	33.39 (4.61)	36.60 (5.83)	**<0.001**
DGLA	1.90 (1.45)	2.09 (0.74)	1,92 (0.61)	0.126
AA	7.70 (1.44)	8.28 (2.92)	8.19 (2.46)	0.534
GLA	0.42 (0.43)	0.41 (0.23)	0.40 (0.27)	0.870
ALA	0.79 (0.78)	0.50 (0.33)	0.43 (0.25)	**0.005**
EPA	0.60 (0.90)	0.27 (0.20)	0.23 (0.14)	**<0.001**
DHA	2.90 (2.35)	1.91 (0.78)	1.19 (0.40)	**<0.001**
DHA/AA	0.4 (0.2)	0.2 (0.1)	0.1 (0.1)	**<0.001**
AA/(EPA+DHA)	2.1 (0.85)	3.9 (1.33)	5.8 (1.45)	**<0.001**
OA/STE	3.1 (1.5)	3.2 (1.1)	2.9 (0.8)	0.473
LA/OA	1.6 (0.8)	1.5 (0.4)	1.7 (0.5)	**0.046**
ALA/LA	0.02 (0.02)	0.02 (0.01)	0.01 (0.00)	**<0.001**
GLA/LA	0.01 (0.02)	0.01 (0.01)	0.01 (0.01)	0.295
AA/DGLA	4.3 (3.6)	4.1 (1.8)	4.2 (2.1)	0.644
EPA/ALA	1.0 (0.9)	0.6 (0.4)	0.6 (0.4)	**0.031**
DHA/ALA	5.80 (5.97)	3.65 (3.11)	2.73 (2.67)	0.146
DHA/EPA	4.8 (5.1)	6.5 (5.5)	5.2 (4.5)	0.164
EPA/AA	0.09 (0.11)	0.04 (0.03)	0.03 (0.02)	**<0.001**
SFA	27.72 (4.17)	26.99 (5.17)	26.65 (3.55)	0.552
MUFA	22.44 (8.18)	23.89 (4.89)	23.08 (5.08)	0.581
PUFA	49.30 (10.12)	47.71 (5.44)	49.60 (4.81)	0.113
PUFA/SFA	1.70 (0.55)	1.75 (0.33)	1.90 (0.40)	0.194
PUFA/MUFA	2.2 (1.0)	2.0 (0.53)	2.1 (0.50)	0.290
(PUFA+MUFA)/SFA	2.6 (0.55)	2.7 (0.63)	2.8 (0.45)	0.578
*n*-6	44.32 (7.07)	44.79 (5.85)	47.88 (4.47)	**0.001**
*n*-3	5.02 (2.40)	2.72 (0.77)	1.94 (0.41)	**<0.001**

Results are expressed as *n* (%) or median (interquartile range). * χ^2^ tests or Kruskal-Wallis tests. Significant differences are in bold.

**Table 5:** Frequency of consumption of foods according to *n*-6/*n*-3 ratio.

Foods	*n*-6/*n*-3 ≤ 10 (*n* = 9)	10 < *n*-6/*n*-3 ≤ 20 (*n* = 58)	*n*-6/*n*-3 > 20 (*n* = 36)	*P*-Value *
*Nuts*				
Almonds	2.0 (1.0)	2.0 (2.0)	2.0 (1.0)	0.264
Cashew nuts	1.0 (1.0)	1.0 (2.0)	1.0 (1.0)	0.583
Hazelnuts	1.0 (1.0)	1.0 (2.0)	1.0 (2.0)	0.130
Peanuts	1.0 (2.5)	1.0 (2.0)	1.0 (2.0)	0.437
Pistachio	1.0 (1.5)	1.0 (1.25)	1.0 (1.75)	0.620
Walnuts	2.0 (2.5)	2.0 (2.0)	2.0 (2.0)	0.836
*Seeds*				
Flax seeds	3.0 (1.5)	1.0 (2.0)	1.0 (1.75)	0.056
Pumpkin seeds	2.0 (2.0)	1.0 (2.0)	1.0 (2.75)	0.079
Sesame seeds	2.0 (1.0)	1.0 (1.0)	1.0 (2.0)	0.415
Sunflower seeds	2.0 (2.0)	1.0 (2.0)	1.0 (2.75)	0.065
*Oils and various*				
Olive oil	5.0 (1.0)	5.0 (1.0)	5.0 (1.0)	0.101
Sunflower oil	<0.001 (1.0)	<0.001 (1.0)	<0.001 (1.0)	0.987
Tahini	1.0 (2.0)	1.0 (2.0)	1.0 (1.75)	0.721
Avocado	2.0 (1.50)	2.0 (2.0)	2.0 (3.0)	0.412
Olives	1.0 (1.50)	1.0 (1.0)	2.0 (1.0)	0.725

Results are expressed as median (interquartile range). Considered categories were: 0 (never), 1 (two to four times a month), 2 (two to three times a week), 3 (four to six times a week), 4 (once a day), and 5 (≥2 times/day). * Pearson’s χ^2^ tests (Flax-seeds *p* = 0.056).
